# Novel regulators of hepatic macrophages in liver fibrosis

**DOI:** 10.3389/fimmu.2025.1705503

**Published:** 2025-12-17

**Authors:** Xiangjun Tang, Meiwen Bai, Xianhong Du, Hong Wang, Meifang Liu, Xiaoyan Fu, Hongxia Zhang, Shujuan Liang, Liyuan Wang

**Affiliations:** Key Laboratory of Immune Microenvironment and Inflammatory Disease Research in Universities of Shandong Province, School of Basic Medical Sciences, Shandong Second Medical University, Weifang, China

**Keywords:** macrophage, liver fibrosis, monocyte recruitment, macrophage polarization, molecular mechanisms

## Abstract

Liver fibrosis is a common pathological process resulting from liver damage and subsequent inflammatory responses in various chronic liver diseases, leading to persistent structural and functional abnormalities in the liver. It can further progress to liver cirrhosis and hepatocellular carcinoma. Currently, no effective treatments are available for liver fibrosis, except for liver transplantation. Hepatic macrophages play essential roles in both the development and regression of liver fibrosis. Understanding the mechanisms by which hepatic macrophages regulate liver fibrosis could identify new therapeutic targets. In this review, we aim to summarize recent discoveries regarding the specific molecular mechanisms underlying the progression of liver fibrosis over the past 5 years, with a special focus on monocyte recruitment and macrophage polarization or differentiation, as well as their roles in disease progression.

## 1 Introduction

Liver fibrosis is a common pathological outcome of chronic liver diseases triggered by various factors, such as hepatitis B virus (HBV) or hepatitis C virus (HCV) infection, alcoholism, metabolic dysfunction-associated steatohepatitis (MASH), cholestasis, and exposure to drugs/toxins. Long-term liver damage leads to abnormal repair, excessive deposition of collagen fibril and extracellular matrix (ECM) from fibrotic scars, and ultimately results in structural disruption and impaired physiological function of liver tissue (1). If left untreated, 2%–6% of patients with liver fibrosis may progress to liver cirrhosis, hepatocellular carcinoma (HCC), and eventually liver failure, causing over one million deaths worldwide annually (2). To date, there are no approved antifibrotic therapies, and liver transplantation remains the only treatment option (3). Therefore, studying the pathological mechanisms and potential therapeutic targets for liver fibrosis is of great significance in both clinical practice and medical research.

Uncontrolled chronic inflammation is a nonnegligible driving force transforming self-limited tissue repair processes into a vicious cycle that boosts the progression of liver fibrosis (4–6). It is worth noting that hepatic macrophages, consisting of resident Kupffer cells (KCs) and monocyte-derived macrophages (MoMFs), are identified as pivotal regulators in initiating, sustaining, and amplifying the inflammatory reaction in acute and chronic liver diseases (7, 8). Upon liver injury, hepatic macrophages recognize multiple stimulants, including damage-associated molecular patterns (DAMPs), mitochondrial DNA (mtDNA), reactive oxygen species (ROS), and pathogen-associated molecular patterns (PAMPs), to generate cytokines and chemokines that amplify the inflammatory response (9–12). In addition, hepatic macrophages secrete transforming growth factor beta (TGF-β) and platelet-derived growth factor (PDGF) to activate hepatic stellate cells (HSCs), promoting their trans-differentiation into collagen-producing myofibroblasts and excessive ECM deposition in the liver (13–16). Different subsets of liver macrophages play distinct roles in the control of fibrosis progression and regression. However, the exact molecular mechanisms of monocyte recruitment, macrophage polarization, and phenotypic switching in fibrotic processes remain unclear. In this review, we aim to highlight the potential molecular mechanisms of hepatic macrophages in the dynamic processes of progression and regression in liver fibrosis over the past 5 years. We further offer insights into the potential mechanisms underlying the phenotypic switch from profibrogenic macrophages to restorative macrophages, the regulation of heterogeneity, and the action modes of hepatic macrophages, with a view to provide theoretical bases and new therapeutic strategies targeting macrophages in liver fibrosis.

## 2 Hepatic macrophages

**Figure 1:**
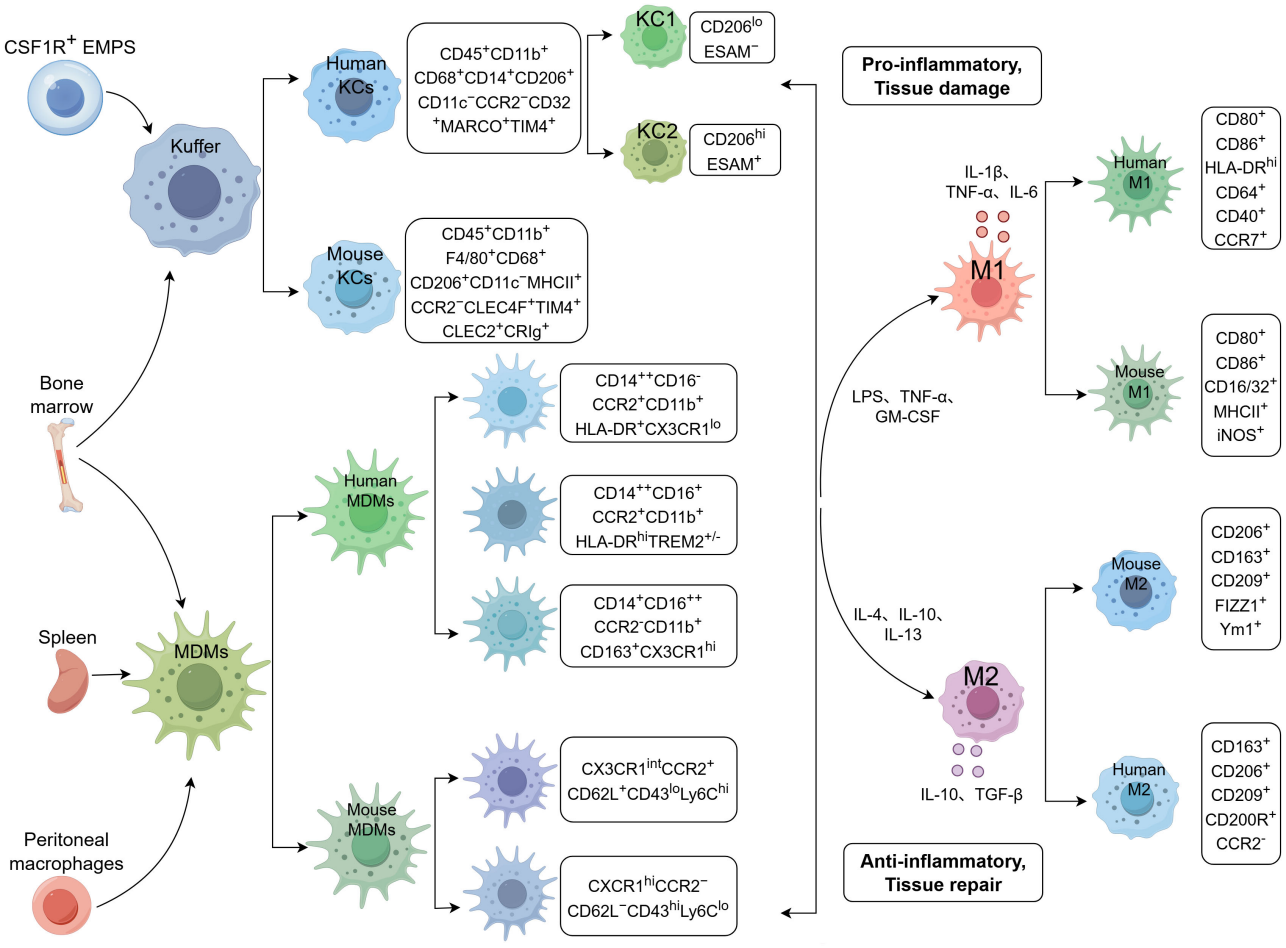
Hepatic macrophage subsets in mice and humans. KCs and MoMFs are the main components of hepatic macrophages. KCs mainly originate from the yolk sac or bone marrow. In humans, KCs exhibit CD45^+^CD11b^+^CD68^+^CD14^+^CD206^+^CD11c^−^CCR2^−^CD32^+^MARCO^+^TIM4^+^ phenotype. In mice, KCs can be marked as CD45^+^CD11b^+^F4/80^+^CD68^+^CD206^+^CD11c^−^MHC II^+^CCR2^−^CLEC4F^+^ TIM4^+^CLEC2^+^CRIg^+^. KCs are further distinguished into KC1 (CD206^lo^ESAM^−^) and KC2 (CD206^hi^ESAM^+^) subgroups in mice. Infiltrating monocytes are mainly derived from bone marrow, spleen, and peritoneal macrophages. The monocytes further differentiate into MoMFs. In mice, MoMFs are categorized into two subsets: CX3CR1^int^CCR2^+^CD62L^+^CD43^lo^Ly6C^hi^ and CX3CR1^hi^CCR2^−^CD62L^−^CD43^hi^Ly6C^lo^ monocytes. In humans, MoMFs are classified into three subtypes: CD14^++^CD16^−^CCR2^+^CD11b^+^human leukocyte antigen DR(HLA-DR)^+^CX3CR1^lo^ (classical), CD14^++^CD16^+^CCR2^+^CD11b^+^HLA-DR^hi^TREM2^+/−^ (intermediate) and CD14^+^CD16^++^ CCR2^−^CD11b^+^HLA-DR^+^CX3CR1^hi^CD163^+^ (nonclassical) monocytes. Classically, macrophages polarize into M1 and M2 subtypes in response to different stimulations. In humans, M1 macrophages exhibit a CD80^+^CD86^+^HLA-DR^hi^CD64^+^CD40^+^CCR7^+^ phenotype, while M2 macrophages represent a CD163^+^CD206^+^CD209^+^CD200R^+^CCR2^−^ phenotype. In mice, M1 subtypes can be identified as CD80^+^CD86^+^CD16/32^+^MHC II^+^iNOS^+^, while M2 can be recognized as CD206^+^CD163^+^CD209^+^FIZZ1^+^Ym1^+^.

Hepatic macrophages are the most abundant immune cells in the liver. Based on their origin, hepatic macrophages can be categorized into resident macrophages and infiltrating macrophages (**Figure 1**) (17). Hepatic resident macrophages, also known as KCs, are the predominant hepatic macrophages in healthy livers and mainly reside in the hepatic sinusoids. KCs originate from yolk sac-derived CSF1R^+^ erythromyeloid progenitors (EMPs), which populate the fetal liver during embryogenesis (18). In mice, KCs specifically express C-type lectin domain family 2 (CLEC2), CLEC4F, complement receptor of the immunoglobulin superfamily (CRIg), and T-cell membrane protein 4 (TIM-4), among others, and can be phenotypically marked as CD45^+^CD11b^+^F4/80^+^CD68^+^CD206^+^CD11c^−^ major histocompatibility complex II (MHC-II)^+^ chemokine receptor 2 (CCR2)^−^CLEC4F^+^TIM4^+^CLEC2^+^CRIg^+^ (19). In humans, KCs exhibit high expression of TIM4 and macrophage receptor with collagenous structure (MARCO), which can be marked as CD45^+^CD11b^+^CD68^+^CD14^+^CD206^+^CD11c^−^CCR2^−^CD32^+^MARCO^+^TIM4^+^ (20). Hepatic macrophages are highly heterogeneous populations undergoing differentiation and polarization into different phenotypes and exerting dual roles during the process of liver fibrosis. Traditionally, macrophages are classified into M1 or M2 subpopulations, as determined by their surface markers or cellular functions (21). Recently, KCs have been further distinguished into two subpopulations based on the expression of CD206 and endothelial cell-selective adhesion molecule (ESAM) in mice. The major subset KC1 (CD206^lo^ESAM^−^) presents an immunological characteristic, while the minor subset KC2 (CD206^hi^ESAM^+^) mainly participates in lipid metabolism pathways (20, 22). Single-cell RNA sequencing (scRNA-seq) and multiplexed error-robust fluorescent *in situ* hybridization (MERFISH) further revealed two macrophage populations visualized in the healthy liver with different patterns of distribution. Cluster 1 is enriched in the periportal area and also dispersed through the lobules, while cells from cluster 2 are scattered more diffusely through the lobules. Both macrophage populations were identified by expression of CD74, while cluster 2 cells express CD5L and MARCO, most consistent with noninflammatory macrophages or KCs. The role of the macrophage populations, however, has not been further explained (23).

Upon liver injury, KCs respond quickly and release cytokines or chemokines to recruit circulating monocytes migrating into the liver, and the infiltrated monocytes further differentiate into MoMFs. MoMFs generally originate from peripheral blood monocytes and, partially, from peritoneal and splenic macrophages. Peritoneal macrophages also contribute to monocyte infiltration. In mice, MoMFs are defined as Ly6C^hi^ and Ly6C^lo^ subgroups according to the expression of Ly6C (24). MoMFs with high expression of Ly6C exhibit proinflammatory and profibrogenic phenotypes. In contrast, MoMFs with low expression of Ly6C promote scar degradation. Further study revealed that CD11b^+^F4/80^+^ macrophages can be further divided into CD11c^−^Ly6C^int^, CD11c^−^Ly6C^hi^, CD11c^+^Ly6C^−^, CD11c^+^Ly6C^int^, and CD11c^+^Ly6C^hi^ subsets, which differ from each other by distinct expression of the chemokine receptors CCR2 and C–X3–C motif chemokine receptor 1 (CX3CR1) (25). In humans, MoMFs can be classified into three subtypes based on the expression of the surface markers CD14 and CD16, a classification recently approved by the Nomenclature Committee of the International Union of Immunological Societies (NOM-IUIS). Classical (CD14^++^CD16^−^) and intermediate monocytes (CD14^++^CD16^+^) are generally considered to have inflammatory properties similar to murine Ly6C^hi^ monocytes in liver fibrosis, whereas nonclassical monocytes (CD14^+^CD16^++^) display patrolling properties similar to murine Ly6C^lo^ monocytes. Both cell types can promote ECM degradation, but CD14^+^CD16^++^ monocytes also exhibit proinflammatory phenotypes (26). Human and mouse inflammatory monocytes express high levels of CCR2 and low levels of CX3CR1, whereas functionally opposing populations show the reverse pattern (27, 28). MoMFs can acquire KC-like phenotypes within hours, driven by multiple signals following acute or specific depletion of KCs in mice (29).

Classically, similar to KCs, MoMFs can differentiate or polarize into M1 and M2 phenotypes (30). Lipopolysaccharide (LPS), tumor necrosis factor α (TNF-α), and granulocyte-macrophage colony-stimulating factor (GM-CSF) can induce macrophage polarization toward the M1 phenotype or classical pathway-activated macrophages. M1 macrophages are a proinflammatory phenotype, characterized by CD80^+^CD86^+^CD16/32^+^iNOS^+^MHC-II^+^, and highly express proinflammatory cytokines or chemokines, such as inducible nitric oxide synthase (iNOS), interleukin (IL)-1, IL-6, and TNF-α, which can result in tissue damage (31). M2 macrophages, known as alternative pathway-activated macrophages, are primarily activated by IL-4, IL-10, and IL-13, and are characterized by specific markers, such as CD206, CD163, CD209, found in inflammatory zone 1 (FIZZ1), Ym1, and others. M2 macrophages function in phagocytosis, immunosuppression, and tissue repair by secreting cytokines, such as IL-10 and TGF-β (32). Under the dynamic microenvironmental signals, M1 and M2 macrophages can change their phenotypes (26).

It is now recognized that the formerly well-established M1/M2 paradigms now appear to be too simple and limited to describe the macrophage activation states. scRNA-seq has shattered this binary, revealing a remarkable heterogeneity and identifying context-specific macrophage subsets in fibrotic livers that do not conform to the classical M1/M2 dichotomy (30, 33). Recent scRNA-seq studies have uncovered a novel scar-associated macrophage (SAM) subset in fibrotic livers. In contrast to resident KCs, SAMs are derived from bone marrow derived macrophages (BMDMs) and are characterized by high expression of triggering receptor expressed on myeloid cells 2 (TREM2) and CD9. Functionally, SAMs are profibrogenic, secreting TGF-β, PDGF, and angiopoietin-like 2 (ANGPTL2), which directly act on HSCs and impair collagen uptake (34–36). Furthermore, in mouse models of liver fibrosis induced by bile duct ligation (BDL) or carbon tetrachloride (CCl_4_), plasminogen receptor KT (Plg-R_KT_) signaling has been implicated in driving the phenotypic transition of macrophages into this profibrotic SAM state (37). Recently identified lipid-associated macrophages (LAMs), presented as TREM2^+^CD9^+^ lysosomal acid lipase (ALIPA)^hi^ apolipoprotein E (APOE)^+^, have been identified as a population central to MAFLD. LAMs exhibit significant overlap with SAMs but demonstrate a unique transcriptional signature enriched for lipid-handling genes. In mouse models of MASH, LAMs are recruited via an oxidized low-density lipoprotein (oxLDL)–TREM2–rapamycin complex 2 (mTORC2) axis and contribute to the resolution of liver fibrosis in a TREM2 and peroxisome proliferator-activated receptors δ/γ (PPAR-δ/γ) dependent manner. Deletion of TREM2 in macrophages impaired injury repair via a lack of debris clearance and consequently increased fibrosis (38). LAMs and LAM-like KCs were also observed in CCl_4_-driven damage, but whether KCs can adopt a LAM phenotype in this model has not been definitively explored.

Cheng et al. described a novel macrophage cluster in CCl_4_-induced mouse models that expresses canonical macrophage markers (e.g., CLEC4F, F4/80, VSIG4) while concurrently coexpressing markers typical of liver sinusoidal endothelial cells (LSECs), such as vascular endothelial growth factor receptor (VEGFR) and EH domain-containing 3 (EHD3). This unique population potentially represents the debated “macrophage-LSEC” hybrid cells and has sparked considerable discussion. Further evidence is required to clarify the function and categorization of this population (39). These findings challenged previous concepts of hepatic cell identity. Key macrophage subsets and their regulatory patterns during liver fibrosis still require further investigation. While scRNA-seq can identify macrophage states, it traditionally loses spatial context. Integrating scRNA-seq with spatial transcriptomics is crucial to address this limitation.

## 3 Dual roles of hepatic macrophages in the progression and regression of liver fibrosis

**Figure 2:**
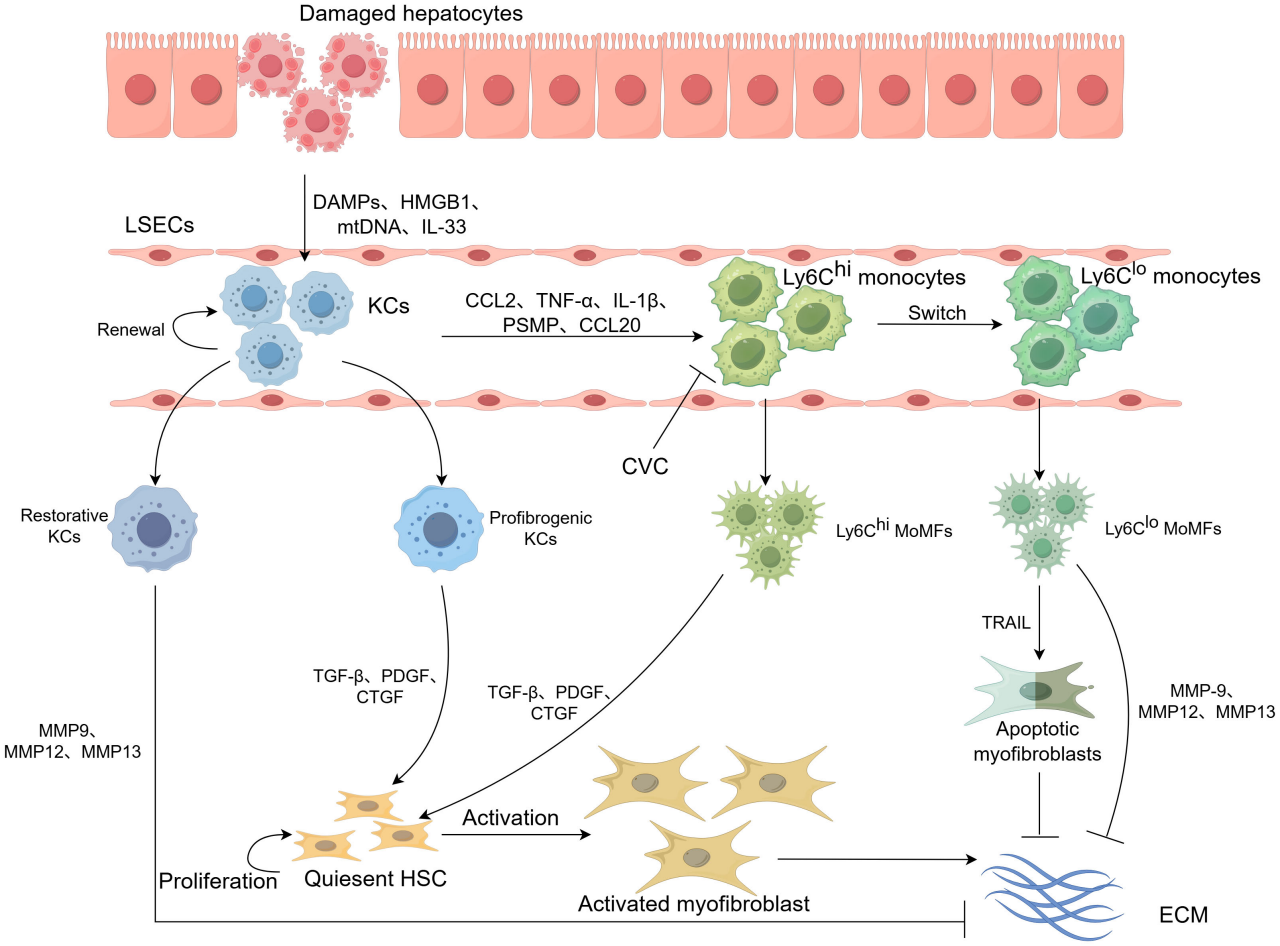
Overview of the roles of hepatic macrophages in liver fibrosis. KC replenishment occurs predominantly by the self-renewal of resident stem cells to maintain liver homeostasis under the physiological conditions. Upon liver injury, KCs are activated by DAMPs, HMGB-1, mtDNA released from damaged hepatocytes, and highly secrete CCL2, TNF-α, IL-1β, and so on, which subsequently contribute to hepatocyte injury and Ly6C^hi^ monocyte infiltration to the damaged site, where they develop into Ly6C^hi^ macrophages in mice. The profibrogenic KCs and Ly6C^hi^ MoMFs promote HSC activation and exert profibrotic effects by expressing TGF-β, PDGF, and CTGF, leading to the excessive deposition of ECM and scar formation. During the regression stage, the KCs and Ly6C^hi^ monocytes undergo phenotype switch and degrade ECM by producing MMPs to exert antifibrotic effects upon phagocytosis or other resolution stimuli.

In general, liver fibrosis is a dynamic, reversible wound-healing process that involves both progression and regression. The transformation of quiescent HSCs into myofibroblasts is central to the pathogenesis of liver fibrosis (13, 40). Hepatic macrophages modulate HSC viability and activation by releasing various cytokines and other factors, thereby playing an important role in both promoting and resolving liver fibrosis (**Figure 2**).

In the early stage of liver injury, hepatic macrophages exert a profibrogenic effect in multiple ways. On one hand, KCs are activated by DAMPs, high mobility group box 1 protein (HMGB-1), mtDNA, and IL-33 released from injured or dead hepatocytes to rapidly secrete a variety of cytokines and chemokines, including C–C motif chemokine ligand 2/monocyte chemoattractant protein-1 (CCL2/MCP-1), TNF-α, IL-1β, to recruit more proinflammatory and profibrogenic monocytes, such as Ly6C^hi^ monocytes in mice and classical monocytes in humans, thereby exacerbating tissue damage (40, 41). The infiltrated MoMFs and KCs release TGF-β, PDGF, and connective tissue growth factor (CTGF) to directly activate HSCs and enhance HSC proliferation via cyclic GMP-AMP synthase (cGAS)-stimulator of interferon genes (STING)/nuclear factor kappa-B (NF-κB) signaling pathways, leading to fibrous scar accumulation at the damaged sites (33, 42). In this stage, inhibiting macrophage activation can reduce the degree of fibrosis.

Once the injury factors are removed, hepatic macrophages switch phenotype and ultimately cause the regression of liver fibrosis (43, 44). Multiple studies have tracked the fate of Ly6C^hi^ monocytes after liver injury has stopped and found that they can transform into Ly6C^lo^CD11b^hi^F4/80^int^ subsets with high expression of matrix metalloproteinases 12/13 (MMP-12/13) and IL-10, termed resolution-phase macrophages. Similarly, proinflammatory KCs switch into anti-inflammatory phenotypes (44). MMPs and their natural inhibitors, tissue inhibitors of metalloproteinases (TIMPs), are critical to regulate ECM remodeling. MMPs are responsible for the degradation of ECM proteins, while TIMPs activate HSCs and inhibit MMP activity to prevent ECM degradation and support HSC survival, thereby promoting fibrosis (45). KCs and Ly6C^lo^ monocytes inhibit TIMP-1 and TIMP-2 production and highly express MMP9 and MMP12/13 to degrade ECM. Ly6C^lo^ monocytes downregulate TGF-β levels and upregulate tumor necrosis factor-related apoptosis-inducing ligand (TRAIL) to stimulate apoptosis of activated HSCs (46). A central unresolved issue is the precise mechanism of macrophage dynamics during fibrosis resolution: whether it is driven primarily by cellular replacement or by phenotypic transdifferentiation.

Overall, the phenotype and functional heterogeneity of hepatic macrophages play critical roles in determining the balance between the mechanisms of progression and resolution of liver fibrosis. Therefore, exploring the key macrophage subsets and molecular mechanisms to control the balance between profibrogenic and restorative macrophage is considered an attractive strategy to promote fibrosis regression (10).

## 4 Mechanisms of hepatic macrophage recruitment and activation during liver fibrosis

### 4.1 Immune microenvironment

The local tissue environment plays a powerful role in recruiting and establishing the distinct macrophage phenotypes (**Figure 2**). Extensive investigations have elucidated that the recruitment and mobilization of circulating monocytes and KCs into injured sites are mediated by diverse cytokines, chemokines, and corresponding receptors. MoMFs can be attracted to the liver via the CCL2/CCR2, CCL5/CCR5, and CCL1/CCR8 chemokine axis, thereby aggravating liver fibrosis (47–49). Inhibiting the recruitment of proinflammatory MoMFs to the liver by interfering with these chemotactic axes is a significant therapeutic strategy. Cenicriviroc (CVC) is a novel antagonist of CCR2/CCR5 with nanomolar potency against both receptors and has been tested in a phase III trial. Hepatic inflammation and fibrosis are significantly improved following a 1-year treatment with CVC in nonalcoholic steatohepatitis (NASH) and liver fibrosis patients (50). In alcohol-fed mice, CVC administration prevents the increase in F4/80^lo^CD11b^hi^ macrophages and reduces proinflammatory Ly6C^hi^ MoMFs in the liver, consequently inhibiting both hepatic inflammation and fibrosis (51). mNOX-E36, a CCL2 inhibitor, can suppress the recruitment of MoMFs to the liver in CCl_4_-induced liver fibrosis models and methionine–choline-deficient diet (MCD)-induced NASH model. Propagermanium (a CCR2 inhibitor) and maraviroc (a CCR5 inhibitor) can also promote the amelioration of NASH in murine models (44). Additionally, IL-33 and its receptor, growth stimulation expressed gene 2 (ST2), can also promote the progression of NASH with fibrosis by regulating CCL2 expression, thereby affecting monocyte recruitment that correlates with collagen expression in mice fed a high-fat diet (HFD) (52, 53).

In addition to the CCL2/CCR2 axis, the CCL20/CCR6 axis can drive hepatic inflammation and fibrosis in patients with alcohol-related steatohepatitis (ASH) and MASH, providing evidence that CCL20/CCR6 mediates inflammatory cell recruitment upon liver injury by promoting infiltration of macrophages and neutrophils (54–56). Deficiency of CCR6 in CCl_4_-treated mice exacerbates liver injury by increasing macrophage recruitment and promoting an M1 phenotype, which results in a worsened inflammatory and fibrogenic liver response to liver injury (57). It has been reported that CCR8 knockout can inhibit the recruitment of MoMFs to the fibrotic liver in CCl_4_- and BDL-treated mice. CCR8-antagonizing peptides (AP8ii) treatment decreases the intrahepatic infiltration of circulating monocytes and attenuates fibrosis in the CCl_4_-induced mouse model (58). In brief, there is significant functional redundancy, and compensatory mechanisms exist among these axes in liver fibrosis. A key knowledge gap is understanding their collaborative or compensatory crosstalk across different disease stages and etiologies. Future research must elucidate this network to develop multitarget or sequential targeting strategies.

ROS are one of the major causes of liver fibrosis, alongside chemokines and cytokines. ROS elimination in the liver inhibits the activation of KCs and macrophage infiltration after CCl_4_ injection in mice (59). During liver injury, dynamin-related protein 1 (DRP1)-mediated mitochondrial fission and Bcl-2/adenovirus E1B interacting protein 3 (BNIP3)-mediated mitophagy activation lead to mitochondrial ROS production in KCs and macrophage activation; this process depends on endocytosis of monomeric Toll-like receptor 4 (TLR4)-myeloid differentiation protein 2 (MD2) complex and NADPH oxidase 2 (NOX2) activation in CCl_4_-/HFD-/LPS-induced hepatic fibrogenesis (60). Autophagy-related gene 5 (ATG5) is a pivotal protein in the assembly of the autophagosome; myeloid-specific deletion of ATG5 leads to increased ROS production and enhanced inflammatory cell recruitment associated with exacerbated liver injury in CCl_4_-induced murine liver fibrosis (61). Downregulation of methyltransferase like 14 (METTL14) reduces the level of glutaminase 2 (GLS2) by affecting the YTH domain family, member 1 (YTHDF1) mediated translation efficiency in an N6-methyladenosine (m6A)-dependent manner, which might contribute to forming an oxidative stress microenvironment and recruiting CX3CR1^+^CCR2^+^ monocytes in HFD-fed mice (62). T-cell immunoglobulin and mucin-domain-containing protein 3 (TIM-3), constitutively expressed on monocytes and macrophages, negatively regulates M1 activation and the secretion of pro-inflammatory cytokines by reducing ROS production, which subsequently attenuates fibrotic responses in the MCD diet-induced liver fibrosis (63). Fibrinogen-like protein 2 (FGL2), markedly increased in the liver tissues of patients with liver cirrhosis and HCV infection, promotes mitochondrial ROS production by interacting with heat shock protein 90 (HSP90) to disrupt protein kinase B (Akt/PKB) phosphorylation as well as downstream forkhead box O1 (FOXO1) phosphorylation. FGL2 augmented mitochondrial ROS production is involved in M1 macrophage polarization that contributes to inflammatory damage and fibrosis development (64). These results suggest the critical role of ROS in macrophage activation during liver fibrosis. Targeting ROS elimination exhibits great potential in liver fibrosis therapy.

### 4.2 Key signaling pathways

**Figure 3:**
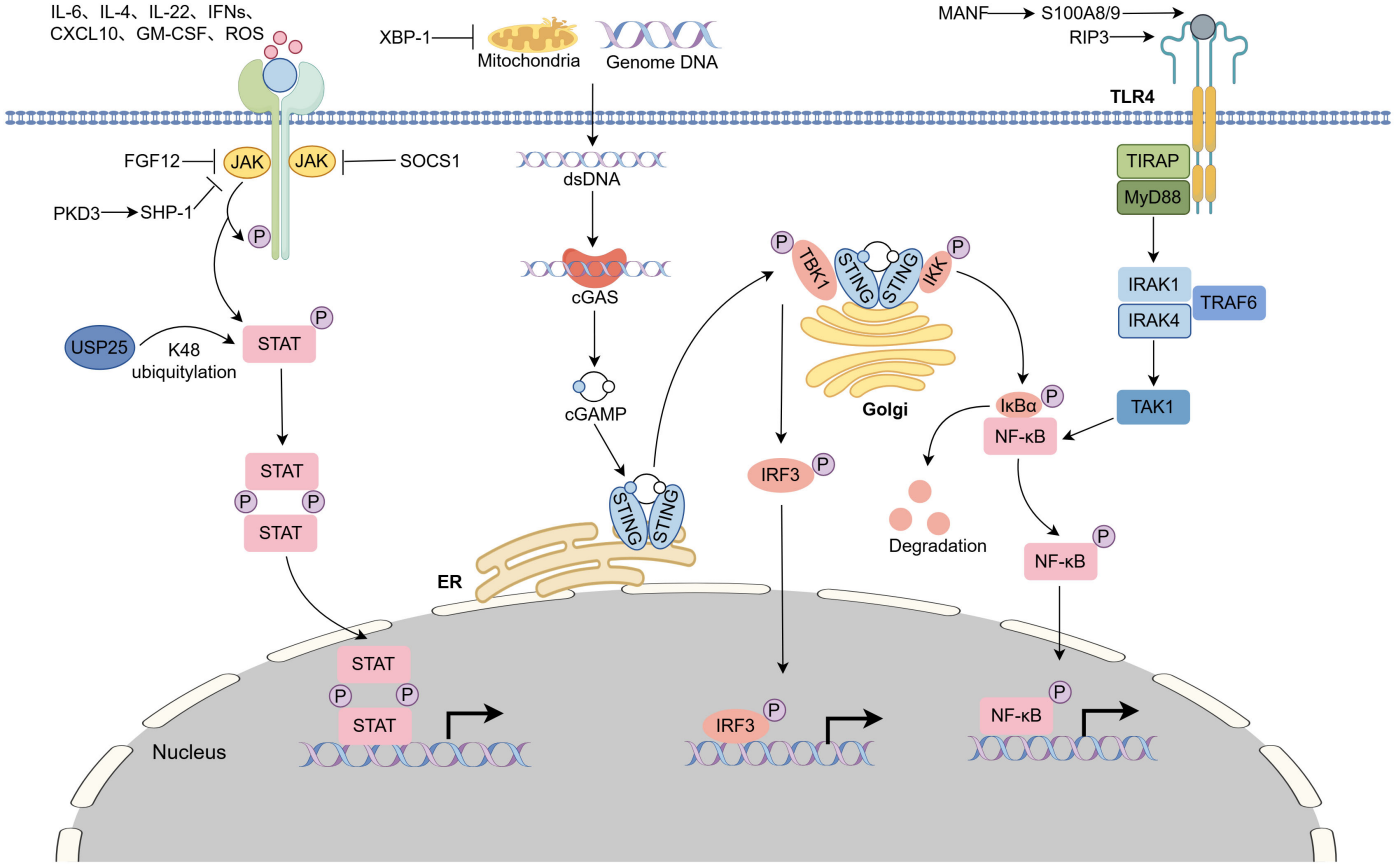
Key signaling pathways involved in hepatic macrophage polarization during liver fibrosis. Hepatic macrophage activation can be regulated by TLR4–MyD88–NF-κB, JAK–STAT, cGAS–STING pathways, etc. (1) TLR4–MyD88–NF-κB, RIP3 interferes with macrophage accumulation by regulating the ROCK1–TLR4–NF–κB signaling pathway. MANF disturbs S100A8/A9 heterodimer-mediated TLR4–NF-κB signaling pathway activation to inhibit M1 macrophage polarization. (2) JAK–STAT pathway, CXCL10 interacting with the receptor CXCR3 negatively regulates macrophages activation depending on SHP-1-mediated STAT6 activation. IL-4Rα regulates alternative macrophage activation in a STAT6-dependent manner. USP25 stabilizes STAT6 by reducing the K48-specific ubiquitination of STAT6. SOCS1 inhibits M1 macrophage polarization by blocking the JAK1/STAT1 pathway. FGF12 induces the Ly6C^lo^ phenotype switch to Ly6C^hi^ by inhibiting the JAK/STAT signaling pathway. IL-22 promotes M2 macrophage polarization by activating the STAT3 pathway. (3) cGAS–STING pathway, cGAS recognizes cytosolic mtDNA to promote STING phosphorylation and macrophage activation. XBP1 deficiency decreases mtDNA release and STING activation by promoting BNIP3-mediated mitophagy.

Many signaling pathways are involved in the initiation, progression, and resolution of liver fibrosis (**Figure 3**). Pattern recognition receptors (PRRs)-mediated inflammatory signaling pathways exert an essential role in macrophage polarization and activation (6, 65). Previous research has revealed that M1 macrophages can be polarized via TLR4/NF-κB and Janus kinase–STAT1 (JAK-STAT1) pathways. M2 macrophages can be polarized via the JAK/STAT6 and TGF-β/Smads signaling pathways (66, 67). Here, we delineate three pivotal pathways governing macrophage activation in the context of liver fibrosis reported in recent years, highlighting their mechanistic underpinnings.

TLRs and their adaptor, myeloid differentiation primary response gene 88 (MyD88), are the key “gatekeepers” of the immune system. Increasing evidence reveals that the TLR–MyD88 pathway plays important roles in liver fibrosis (68). MyD88 deficiency in myeloid cells reduces macrophage activation and the secretion of CXCL2 and TGF-β, thereby restraining HSC activation in CCl_4_-treated mice (69, 70). TLR signaling does not operate in isolation. Receptor-interacting protein kinase-3 (RIP3) is essential for necroptosis. The absence of RIP3 in macrophages alleviates macrophage and neutrophil accumulation by regulating the Rho‐associated coiled‐coil containing protein kinase 1 (ROCK1)–TLR4–NF-κB signaling pathway in mice after CCl_4_ or BDL treatment (71). Mesencephalic astrocyte-derived neurotrophic factor (MANF) is an endoplasmic reticulum (ER) stress-inducible protein that is extensively expressed in the liver. Hepatic inflammation upregulates MANF expression and secretion, which preferentially interacts with S100A8 to form the S100A8/MANF complex and disrupts the formation of the S100A8/A9 heterodimer. This disturbance blocks S100A8/A9-mediated TLR4–NF-κB pathway activation, subsequently promoting the differentiation of monocytes into Ly6C^lo^ macrophages. Myeloid-specific MANF knockout increases the population of hepatic Ly6C^hi^ macrophages and exacerbates CCl_4_-induced hepatic fibrosis in mice (72).

Many cytokines and growth factors are involved in liver fibrosis through the JAK–STAT pathway. Inhibition of STAT1 phosphorylation in macrophages can suppress M1 macrophage polarization (73). CXCL10, interacting with its receptor CXCR3, activates the JAK/STAT1 pathway, contributing to macrophage polarization and CCl_4_-induced murine liver fibrosis (74). Suppressor of cytokine signaling 1 (SOCS1), an indispensable regulator of IFN-γ signaling, regulates macrophage polarization toward the M1 phenotype through the JAK1/STAT1 pathway in mice with liver fibrosis and cirrhosis challenged with CCl_4_ (75). Fibroblast growth factor 12 (FGF12) induces a macrophage phenotype switch from Ly6C^lo^ to Ly6C^hi^ by inhibiting JAK/STAT signaling pathways in BDL- and CCl_4_-induced liver fibrosis (76). During reversal, macrophage IL-4Rα signaling transcriptionally activates the expression of fibrotic MMPs, especially MMP-12, to regulate alternative macrophage activation in a STAT6-dependent manner in mice treated with CCl_4_ and an MCD diet (77). Ubiquitin-specific peptidases 25 (USP25) reduces the K48-specific ubiquitination of STAT6, thereby promoting IL-4-induced anti-inflammatory polarization of hepatic macrophages and BDL-induced liver fibrosis (78). Protein kinase D3 (PKD3) deficiency decreases the phosphatase activity of SH2-containing protein tyrosine phosphatase-1 (SHP-1), resulting in hepatic macrophage polarization toward a profibrogenic phenotype depending on sustained STAT6 activation in mice with CCl_4_ treatment (79). IL-22 exerts anti-inflammatory effects by polarizing KCs to M2 phenotype via activation of the signal transducer and activator of transcription 3 (STAT3) pathway and suppression of extracellular-regulated kinase 1/2 (ERK1/2) and Akt pathways in CCl_4_-induced liver fibrosis (80). Previous studies lack a precise delineation of the JAK–STAT signaling network within specific hepatic macrophage subsets and at specific time points.

cGAS, a cytosolic double-stranded DNA (dsDNA) sensor, and its downstream effector, STING, are important for the innate immune response to intracellular DNA in liver diseases (81, 82). Notably, STING expression in MoMFs is associated with the progression of liver inflammation and fibrosis in patients with nonalcoholic fatty liver disease (NAFLD) and HBV infection (83, 84). mtDNA leaked from damaged hepatocytes and activated KCs stimulates STING to regulate macrophage activation; myeloid cell-specific STING disruption reduces the severity of HFD- and MCD-induced NASH (81). X-box binding protein 1 (XBP1) is a factor of endoplasmic reticulum (ER) stress signaling pathways. XBP1 deficiency decreased the cytosolic mtDNA release and STING/NLR family pyrin domain containing 3 (NLRP3) activation by promoting BNIP3-mediated mitophagy activation in macrophages. Pharmacological inhibition of XBP1 ameliorates CCl_4_ injection-, BDL-, or MCD diet-induced liver fibrosis progression in mice (85). However, it remains unclear whether these signaling pathways operate in parallel or interact with each other during the pathogenesis of hepatic fibrosis.

### 4.3 Noncoding RNA

**Figure 4:**
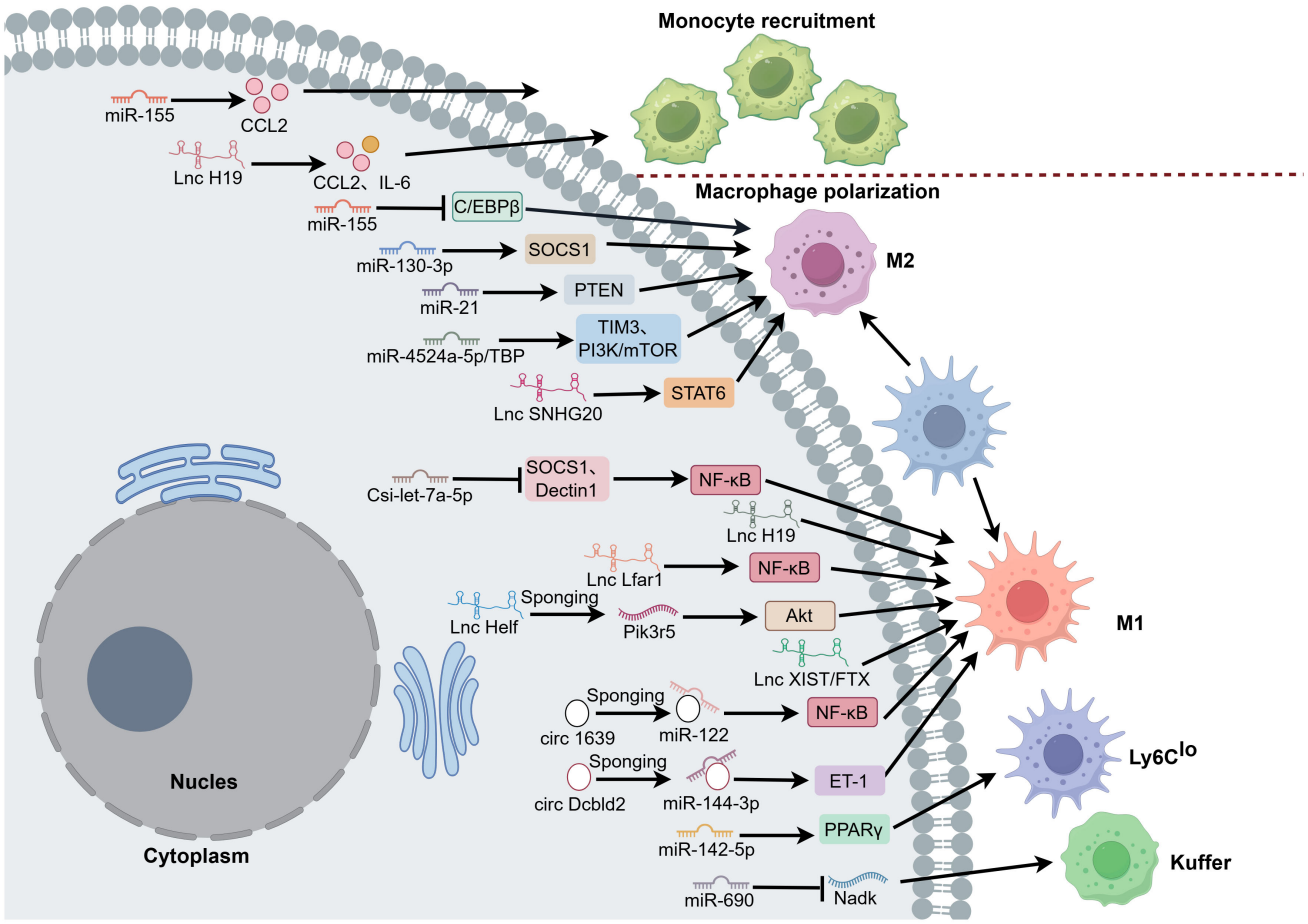
ncRNAs regulate the adhesion and activation of hepatic macrophages during liver fibrosis. The ncRNAs, including miRNAs, lncRNAs, and circRNAs, play important roles in the progression of liver fibrosis. (1) miRNAs, miR-155 promotes CCL2 expression to induce monocyte infiltration and influences M2 macrophage polarization by decreasing C/EBPβ expression. MiR-130a-3p, cooperating with miR-142-5p, controls macrophage polarization to the M2 phenotype and Ly6C^lo^ phenotype by targeting SOCS1 and PPAR-γ, respectively. MiR-21 negatively regulates M2 polarization by targeting PTEN. MiR-4524a-5p/TBP increased M2 macrophage polarization via the TIM3 and PI3K/mTOR pathways. MiR-690 maintains KC functions by downregulating *Nadk* transcription. Csi-let-7a-5p promotes M1 macrophage activation by blocking SOCS1- and Dectin1-mediated NF-κB signaling pathway. (2) lncRNAs, H19 enhances the M1 polarization of Kupffer cells and promotes the macrophage recruitment via CCL2 and IL-6. Lfar1 negatively regulates M1 macrophage activation through the NF-κB pathway and NLRP3 inflammasome-mediated proptosis. Helf, interacting with PTBP1, increases *Pik3r5* mRNA stability and activates the AKT pathway to promote hepatic macrophage polarization to M1 phenotype. XIST/FTX also increases M1 macrophage polarization. SNHG20 induces M2 polarization through activating STAT6. (3) CircRNAs, Circ_1639 contributes to NF-κB pathway-mediated Kupffer cell activation by sponging miR-122 and regulating TNFRSF13C expression. CircDcbld2 promotes macrophage activation by binding miR-144-3p and regulating Et-1 expression.

Recently, many studies have revealed that non-coding RNAs (ncRNA), including microRNA (miRNA), long non-coding RNA (lncRNA), and circular noncoding RNAs (circRNAs), play important roles in the occurrence and progression of liver fibrosis (**Figure 4**).

#### 4.3.1 MiRNAs

MiRNAs, a class of small noncoding endogenous RNA molecules that regulate gene expression through translation repression or mRNA degradation, have distinct roles in modulating macrophage polarization, tissue infiltration, and inflammation regression (86). For instance, miR-155 depletion inhibits monocyte infiltration by suppressing CCL2 expression, and KCs exhibit a predominance of the M2 phenotype due to increased CCAAT enhancer-binding proteinβ (C/EBPβ) expression during liver fibrosis associated with NASH and ASH (87, 88). Furthermore, in a CCl_4_-induced murine model, miR-130a-3p cooperates with miR-142-5p to control macrophage polarization by targeting SOCS1 and PPAR-γ pathways, respectively, thereby promoting M2 macrophage polarization in the murine liver fibrosis model induced by CCl_4_ (89). This M2-promoting role of miR-130a-3p, which induces a Ly6C^lo^ macrophage phenotype, is also observed in schistosomiasis-induced fibrosis (90). Similarly, miR-21 deficiency promotes M2 polarization by targeting phosphate and tension homology deleted on chromosome 10 (PTEN) in mice exposed to arsenite (91). MiR-4524a-5p/TATA box binding protein (TBP) increases β-transducin repeat containing protein (β-TrCP)-mediated TIM3 polyubiquitination and membrane translocation. The ubiquitinated TIM3 then binds with phosphatidylinositol 3-kinase (PI3K), followed by inhibition of mammalian target of rapamycin (mTOR), which ultimately contributes to M2 macrophage polarization and aggravates NAFLD-associated fibrosis (92).

Extracellular vesicles (EVs), particularly exosomes, are established mediators of intercellular communication (93). MiRNAs can be packaged into exosomes to affect the process of liver fibrosis. For example, EV-encapsulated miR-690 from KCs can maintain specific KC functions by downregulating NAD^+^ kinase (*Nadk*) mRNA levels and lead to reduced fibrosis in a mouse model of NASH (94). MicroRNA Csi-let-7a-5p delivered by EVs from liver fluke can drive the activation of M1 macrophages, which further contributes to *Clonorchis sinensis*-caused fibrosis by blocking SOCS1 and DC-associated C-type lectin 1 (Dectin1)-mediated NF-κB signaling pathway (95).

#### 4.3.2 LncRNAs

Emerging evidence has established the critical roles of long noncoding RNAs (lncRNAs) in regulating macrophage differentiation and apoptosis (96). Several specific lncRNAs have been implicated in macrophage polarization during liver fibrosis. For instance, cholangiocyte-derived exosomal lncRNA H19 is closely correlated with macrophage activation and hepatic fibrosis in BDL cholestatic mouse models, as well as in human primary sclerosing cholangitis (PSC) and primary biliary cholangitis (PBC) patients. H19 can enhance the M1 polarization of KCs and promote the recruitment and differentiation of BMDMs via CCL2 and IL-6 (97). Silencing lncRNA Lfar1 alleviates CCl_4_- and BDL-induced M1 macrophage activation through the NF-κB pathway and NLRP3 inflammasome-mediated proptosis (98). Another profibrotic lncRNA, Helf, interacts with RNA-binding protein polypyrimidine tract binding protein 1 (PTBP1) to promote its interaction with phosphoinositide-3-kinase regulatory subunit 5 (*Pik3r5*) mRNA, resulting in increased stability and activation of the Akt pathway, thus promoting hepatic macrophage polarization to M1 phenotype and liver fibrosis progression caused by CCl_4_ or BDL treatment (99). On the other hand, some lncRNAs exert antifibrotic effects. Downregulation of lncRNA XIST/FTX, which is elevated in HFD-induced metabolic dysfunction-associated steatotic liver disease (MASLD) mice, promotes KC M2 polarization (100). Similarly, small nucleolar RNA host gene 20 (SNHG20) induces M2 polarization through activating STAT6, thereby delaying the progression from NALFD to HCC in mice (101).

#### 4.3.3 CircRNAs

CircRNAs are key regulators of biological processes, functioning as miRNA sponges, translators of peptides, and scaffolds for proteins or RNA (102). In liver fibrosis, the expression profile of circRNAs is significantly dysregulated compared to normal controls, and these molecules are closely implicated in the activation of HSCs, macrophage-driven inflammation, and oxidative stress (103–105). For instance, Circ_1639 is upregulated in Kupffer cells of ethanol-fed mice, where it activates the NF-κB pathway and promotes proinflammatory factor production by sponging miR-122 and elevating tumor necrosis factor receptor superfamily member 13C (TNFRSF13C) expression in alcoholic liver disease (106). Similarly, in a CCl_4_-induced fibrosis model, CUB and LCCL domain-containing 2 (Dcbld2) originating from KCs promotes macrophage activation and oxidative stress. The stability of circDcbld2 is enhanced through WT1-associated protein (WTAP)-mediated m6A methylation, which is recognized by insulin-like growth factor 2 mRNA-binding protein 2 (IGF2BP2). Furthermore, circDcbld2 competitively binds to miR-144-3p, thereby derepressing its target endothelin-1 (Et-1) mRNA. The subsequent upregulation of Et-1 expression further amplifies macrophage-mediated inflammation and oxidative stress, driving the progression of liver fibrosis (107).

Overall, current investigations into ncRNAs remain largely insular, lacking comprehensive integration of network topology within miRNA-associated signaling pathways. Consequently, this gap impedes the prediction of interventional efficacy from a systems-level perspective.

### 4.4 Interacting with other cells

**Figure 5:**
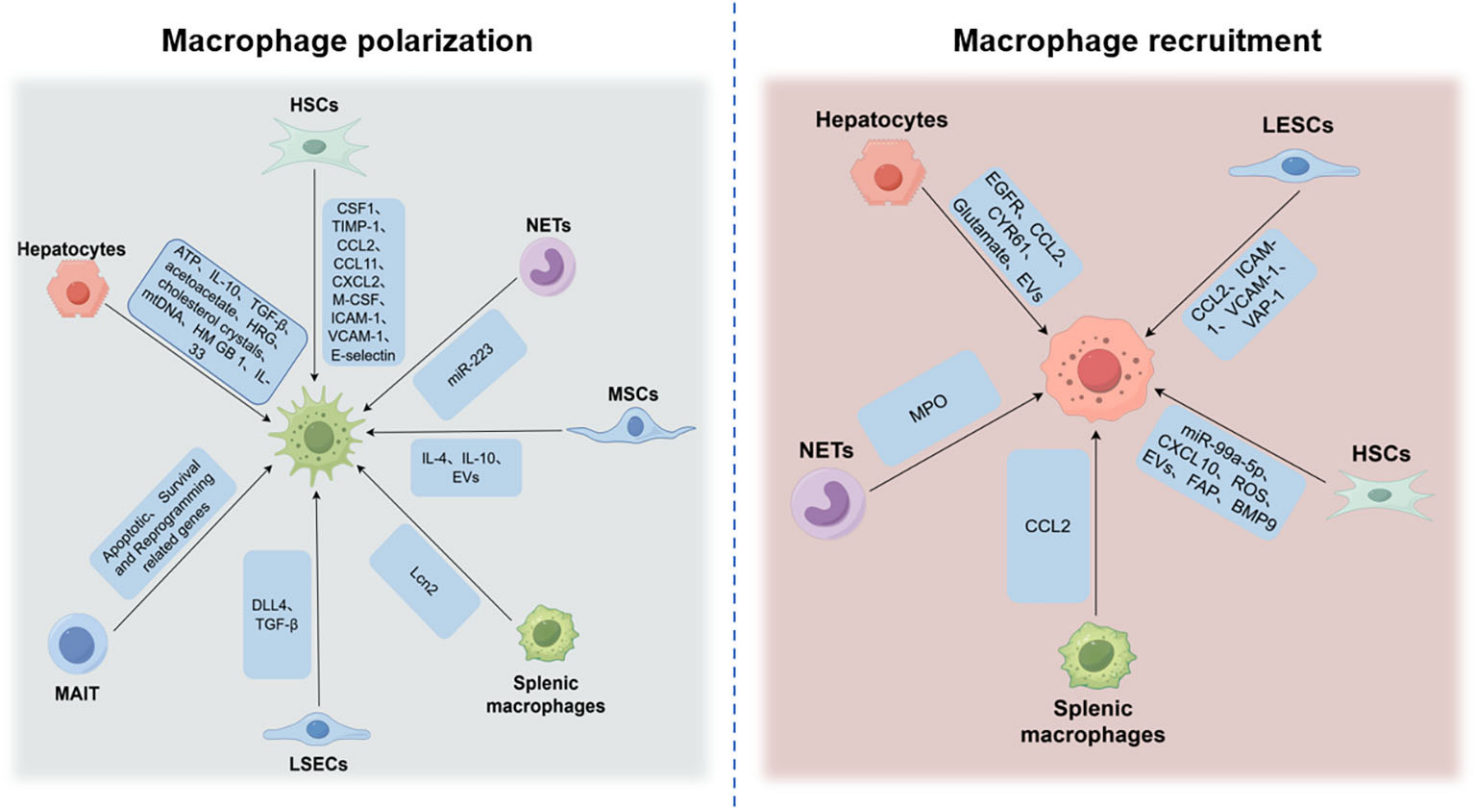
Cellular crosstalk between hepatic macrophages and surrounding cells in hepatic fibrosis. The crosstalk between hepatic macrophages and other cells in the liver plays an important role during the hepatic fibrosis process. The ATP, IL-10, TGF-β, acetoacetate, HRG, cholesterol crystals within remnant lipid droplets, and DAMPs, including mtDNA, HMGB1, IL-33, etc., derived from damaged or dead hepatocytes, contribute to macrophage polarization. EGFR, CCL2, PSMP, CCL7, CYR61, glutamate, and EVs from hepatocytes are required for monocyte recruitment. Activated HSCs can release CSF1, TIMP-1, CCL2, CCL11, CXCL2, M-CSF, ICAM-1, VCAM-1, E-selectin, etc., which can promote macrophage recruitment. HSC-derived miR-99a-5p, CXCL10, ROS, EVs, and HSC-associated proteins such as FAP and BMP9 exert important roles in hepatic macrophage polarization. LSECs facilitate hepatic recruitment of monocytes by expressing CCL2, ICAM-1, VCAM-1, and VAP-1. DLL4 and TGF-β secreted by LSECs can induce monocytes to acquire and maintain Kupffer cell identity. MPO is associated with monocyte infiltration and hCLS formation. NETs stimulate macrophage pyroptosis. Neutrophils induce alternative activation of macrophages, potentially via miR-223. MSC-derived IL-4, IL-10, and exosomes containing miR-148a can regulate macrophage phenotype. Activated MAIT can switch macrophage phenotypes by regulating apoptotic, survival, and reprogramming-related genes. Splenic macrophage-secreted CCL2 induces the infiltration of circulating monocytes. Spleen-derived Lcn2 contributes to the infiltrated MoMF and KC activation. Hence, interfering with intercellular communication is a potential therapeutic strategy for liver fibrosis.

Liver lobules are composed of parenchymal and nonparenchymal cells. Hepatocytes are the primary component of hepatic lobules, and nonparenchymal cells (NPCs) constitute 35% of liver cells in the hepatic sinusoids, including LSECs (50%), KCs (20%), lymphocytes (25%), biliary cells (5%), and HSCs (< 1%). It has been suggested that the crosstalk between different cells plays a critical role in the development and progression of liver fibrosis. Here, we focus on the interaction between hepatic macrophages and other cells in the liver (**Figure 5**).

#### 4.4.1 Hepatocytes and macrophages

It is now generally recognized that the damaged hepatocyte plays an active role in fibrogenesis. It contributes to KC activation and MoMF recruitment by releasing a diverse array of DAMPs, including cytosolic proteins, purine nucleotides, and mtDNA (108, 109). For example, high mobility group box 1 protein (HMGB1), a widely studied DAMP, can induce NF-κB translocation and cytokine release by binding TLRs, as evidenced by KCs in NAFLD, HCC, and hepatic ischemia/reperfusion injury (IRI) murine models (110). Conversely, hepatocyte HSPA12A has been identified as a key inhibitor of IRI, functioning by blocking a critical pathway of glycolysis-driven HMGB1 lactylation and secretion, which is essential for macrophage chemotaxis and activation (111). Similarly, mtDNA released from injured hepatocytes can activate the TLR9 and STING pathways in KCs, exacerbating NAFLD progression (9–11, 112). Injured hepatocytes can release adenosine triphosphate (ATP) into the extracellular space, which can be sensed by P2X purinoceptor 7 (P2X7) receptor and mediate KC activation via the NLRP3 inflammasome in the context of NAFLD (113). Cholesterol crystals within remnant lipid droplets of dead hepatocytes activate the NLRP3 inflammasome in KCs and cause a proinflammatory response. NLRP3 inflammasome blockade improves cholesterol crystal-induced inflammation and fibrosis in NASH models (114). However, the dynamic interplay and potential hierarchy between these parallel DAMP–PRR axes in shaping the overall macrophage response remain unclear.

As mentioned above, the CCL2/CCR2 chemokine axis is a well-established master regulator of monocyte recruitment to the injured liver. Notably, hepatocyte-intrinsic pathways play a pivotal role in initiating this chemokine cascade. For instance, SOCS1 in hepatocytes acts as a negative regulator, inhibiting CCL2 expression and subsequent monocyte infiltration to alleviate CCl_4_-induced hepatic fibrosis (115). Conversely, heightened Notch signaling in hepatocytes, which correlates with NASH severity in patients, drives CCL2 secretion and promotes the infiltration of MoMFs in NASH (116). Intriguingly, while the CCL2/CCR2 axis primarily recruits and polarizes macrophages toward a proinflammatory M1 state, hepatocyte-derived Notch ligands can promote M2 polarization of macrophages, which is reversible upon γ-secretase inhibition (117, 118). This indicates that the net effect on the macrophage landscape is determined by the integration of multiple, potentially opposing, signals from the injured hepatic microenvironment. CCR2 is not only a high-affinity receptor for CCL2, but CCL7 can also bind to CCR2 with high affinity (47). CCL7 expression is inducible in hepatocytes in both an LPS-induced acute liver injury model and a MCD diet-induced chronic liver injury model. Hepatocyte-specific deletion of Brahma-related gene 1 (BRG1), a chromatin remodeling protein, results in a loss of CCL7 induction and subsequently reduces macrophage infiltration in murine livers (118). PC3-secreted microprotein, or microseminoprotein (MSMP/PSMP), is a novel chemotactic cytokine that functions as a high-affinity ligand for CCR2. Upon liver injury, hepatocytes are stimulated by DAMPs such as HMGB-1 and IL-33 to produce PSMP, which subsequently fuels a profibrotic cascade by driving inflammatory macrophage infiltration, polarizing macrophages toward an M1 phenotype, and directly activating HSCs. The therapeutic potential of targeting this axis is underscored by the finding that a PSMP-neutralizing antibody (3D5) significantly ameliorates fibrosis in three murine models, including mice treated with CCl_4_, BDL, or a 5-diethoxycarbonyl-1,4-dihydrocollidine diet (DDC). PSMP also directly promotes M1 macrophage polarization *in vitro* (119).

Beyond direct cell–cell contact, hepatocytes orchestrate hepatic macrophage biology through a diverse secretome, comprising metabolites, proteins, and EVs. For instance, metabolites like acetoacetate from hepatocytes in HFD-fed mice can be metabolized by macrophages via succinyl-coenzyme A-oxoacid transferase (SCOT), potentially supporting the metabolic programs of alternatively activated macrophages (120). Conversely, liver-derived histidine-rich glycoprotein (HRG) appears to be an endogenous molecular factor promoting polarization of hepatic macrophages toward the M1 phenotype, thereby promoting chronic liver injury and fibrosis progression induced by CCl_4_ and MCD diet (121). The hepatocyte-secreted matrikine cysteine-rich angiogenic inducer 61 (CYR61)/cellular communication network factor 1 (CCN1) promotes monocyte recruitment and their polarization into pro-fibrotic phenotypes via an interleukin-1 receptor-associated kinase 4 (IRAK4)/spleen tyrosine kinase (SYK)/NF-κB-dependent cascade in NASH (122). Similarly, lipotoxic hepatocyte-derived EVs, enriched with active integrin β1 (ITGβ1), promote monocyte adhesion and liver inflammation in murine NASH models (123). Hepatocytes release EVs through the death receptor 5 (DR5) upon fatty acid palmitate stimulation, driving proinflammatory polarization of BMDMs toward proinflammatory phenotypes depending on the ROCK1 pathway in the fructose-fat-cholesterol (FFC) diet mouse models (124). Pericentral hepatocytes can respond to liver injury through glutamate-mediated paracrine signaling, which is controlled by unconventional RPB5 prefoldin interactor1 (URI1). Loss of URI1 in hepatocytes reduces glutamine synthase (GS) activity, increasing circulating glutamate levels and thereby influencing monocyte migration to the injured site and activation (125). The release of iron from macrophages is tightly regulated by the interaction between the peptide hormone hepcidin, produced by hepatocytes, and the macrophage iron exporter ferroportin (FPN1). FPN1 downregulation in hepatocytes promotes macrophage proliferation and polarization toward an M2-like phenotype and liver fibrosis by inducing IL-10 and TGF-β expression *in vivo* and *in vitro* (126). Furthermore, the epidermal growth factor receptor (EGFR) and its interactive signaling partner Erb-B2 receptor tyrosine kinase 3 (ERBB3) axis in hepatocytes is essential for the recruitment of profibrotic Ly6C^hi^ monocytes in CCl_4_-induced liver fibrosis (127).

#### 4.4.2 HSCs and macrophages

Activated hepatic stellate cells (HSCs) are now recognized not only as the primary ECM-producing cells but also as active architects of the hepatic immune microenvironment, critically regulating macrophage recruitment, retention, and functional polarization through a multifaceted signaling network (128). Activated HSCs can modulate the functions of macrophages via a series of chemokines (e.g., CCL2, M-CSF) in murine NASH models (128). It is well-established that macrophage CSF1 is the principal regulator of macrophage biology, orchestrating their differentiation, survival, proliferation, and homeostatic renewal (129). HSCs are the key cellular source of CSF1 and CCL2 in the liver (130). ASH1-like histone lysine methyltransferase (ASH1L)-mediated H3K4 methylation (H3K4me3) increases the expression of CCL2 and CSF1 in HSCs, thereby enhancing their transcription and facilitating macrophage infiltration and polarization in diethylnitrosamine (DEN)/CCl_4_-induced models (131). Beyond its role in ECM homeostasis, TIMP-1 secreted by HSCs serves as a proinflammatory mediator. It epigenetically represses miRNA-145, leading to the de-repression and subsequent upregulation of the transcription factor friend leukemia virus integration 1 (Fli-1) under CCl_4_ treatment. Elevated Fli-1 levels then function as a direct transcriptional driver of *Ccl2* gene expression, ultimately fueling the recruitment of profibrotic macrophages (132). Similar to hepatocytes, ablation of SOCS1 in HSCs displayed heightened infiltration of Ly6C^hi^CCR2^+^ proinflammatory macrophages via regulation of CCL2 expression in CCl_4_-induced models (133). Moreover, MyD88 signaling deficiency in HSCs leads to dysregulation of macrophage polarization triggered by CCl_4_, which is characterized by diminished expression of both CD86 and CD206. This altered polarization phenotype is primarily attributed to an impaired production of CXCL10 (74, 134). HSCs can also induce the migration of KCs by secreting the intercellular adhesion molecule 1 (ICAM-1), vascular cell adhesion molecule 1 (VCAM-1), and E-selectin in response to lipopolysaccharide (LPS) stimulation, thereby solidifying their role as a central hub in coordinating the innate immune response in the fibrotic liver (135). As mentioned before, ROS serve as critical driving forces in the activation and functional polarization of macrophages, shaping their inflammatory and immune responses. Peroxidasin (PXDN) deficiency in hepatic stellate cells (HSCs) is implicated in the accumulation of reactive oxygen species (ROS), thereby promoting macrophage recruitment and skewing their polarization toward a proinflammatory phenotype via the NF-κB pathway in mouse models of liver fibrosis induced by CCl_4_ or a choline-deficient, l-amino acid-defined, high-fat diet (CDAHFD) (136). Pharmacological inhibition of fibroblast activation protein (FAP) in HSCs attenuates the infiltration and activation of macrophages via dampening their inflammatory activation and modulating the expression of matrix-remodeling factors such as MMP in liver fibrosis models induced by CCl_4_ (137). In addition to its well-established role in macrophage survival and inflammation, liver X receptor (LXR) signaling is subject to paracrine regulation. Bone morphogenetic protein 9 (BMP9) released from HSCs acts in concert with Notch signaling to potentiate LXRα activation, a mechanism that controls the KC module upon LPS challenge *in vitro* (130).

EVs originating from long-term activated HSCs (14d) induce BMDM toward an alternative phenotype, whereas short-term activated HSCs (3d) induce the KCs toward a classical M1 phenotype. HSC-derived miR-99a-5p negatively regulates the alternative polarization of macrophages and further disrupts collagen deposition in a CCl_4_-induced liver injury mouse model (138). A critical question is how HSCs in different activation periods differentially modulate macrophage phenotype through distinct secretory profiles.

#### 4.4.3 LSECs and macrophages

LSECs, the most abundant NPCs, are the gatekeepers of the liver, maintaining hepatic homeostasis. Their phenotypic switch during injury to a proinflammatory state initiates a critical dialogue with hepatic macrophages, a crosstalk now recognized as a fundamental driver of liver fibrosis pathogenesis and a promising therapeutic frontier (139). Upon injury, LSECs undergo capillarization and adopt a pro-inflammatory phenotype. A pivotal mechanism in this process is the robust production of CCL2, which orchestrates the recruitment of CCR2-expressing monocytes from the circulation, thereby amplifying the local inflammatory cascade in response to LPS stimulation (140, 141). This transcriptional activation of *Ccl2* is enhanced by the epigenetic regulator p300. p300, interacting with NF-κB and bromodomain-containing protein 4 (BRD4), leads to H3K27 acetylation at the *Ccl2* enhancer to activate *Ccl2* transcription in LSECs, which subsequently promotes macrophage accumulation in CCl_4_ or partial inferior vena cava ligation (pIVCL) models (142). Furthermore, in the context of NASH, LSECs upregulate an array of adhesion molecules—including ICAM-1, VCAM-1, and vascular adhesion protein-1 (VAP-1)—thereby facilitating adhesion and trans-endothelial migration of monocytes, which subsequently differentiate into proinflammatory macrophages (143). While we understand that LSECs recruit monocytes, the functional consequence of this recruitment on macrophage differentiation and polarization is not fully delineated.

Moreover, LSECs can orchestrate the repopulation of the KC niche by instructing recruited monocytes to adopt a “KC phenotype” within days, as revealed upon conditional KC depletion (10). This transdifferentiation is driven by LSEC-derived signals, notably delta-like ligand 4 (DLL4) and TGF-β. DLL4-mediated activation of the Notch transcriptional effector recombination signal binding protein for immunoglobulin kappa J region (RBPJ) activates poised enhancers, leading to the induction of KC lineage-determining factors such as LXRα. These factors reprogram the repopulating liver macrophage enhancer landscape to converge on that of the original resident KCs, which orchestrate monocyte engraftment and imprinting of the KC phenotype (130, 144). These findings provide a fundamental framework for understanding tissue-specific macrophage imprinting. However, their relevance in disease contexts, particularly liver fibrosis, represents a significant knowledge gap. It is imperative to determine whether LSECs retain their instructive capacity amidst fibrotic remodeling and whether profibrotic stimuli alter this phenotypic switching to foster a maladaptive macrophage population that perpetuates disease.

#### 4.4.4 Other cells and macrophages

Intricate cooperation between neutrophils and macrophages can also orchestrate the resolution of inflammation and tissue repair (145). Beyond their classical proinflammatory role, neutrophils are instrumental in guiding macrophage polarization toward a proresolving phenotype, thereby facilitating tissue repair. A key mechanism involves neutrophil-derived ROS, which drive the phenotypic conversion of proinflammatory Ly6C^hi^CX3CR1^lo^ macrophages to proresolving Ly6C^lo^CX3CR1^hi^ macrophages, triggering liver repair (146). This restorative switch is further supported by neutrophil-derived miR-223 in models of CCl_4_- and MCD diet-induced fibrosis (147). Conversely, specific neutrophil activation states can exacerbate disease. For instance, NLRP3 inflammasome activation within neutrophils is a pivotal event in sterile liver injury, critically orchestrating macrophage recruitment and amplifying the progression of inflammation and fibrosis (146). Furthermore, the formation of hepatic crown-like structure (hCLS), a distinctive histological entity in NASH, is orchestrated in part by neutrophil-derived myeloperoxidase (MPO). Critically, neutrophil extracellular traps (NETs) potentiate the progression of chronic liver inflammation and fibrosis by stimulating absent in melanoma 2 (AIM2) inflammasome-mediated pyroptosis in macrophages, as demonstrated in biopsy tissue and blood specimens from chronic hepatitis patients and *in vitro* (148, 149). Although the complex and dual roles of LSECs in liver fibrosis are increasingly recognized, the precise molecular and environmental cues that determine whether neutrophils will adopt a proresolving or profibrotic phenotype are not fully defined.

Mesenchymal stem cells (MSCs) have demonstrated considerable efficacy in promoting the regression of liver fibrosis, primarily through their ability to reprogram hepatic macrophages. Evidence from CCl_4_-induced cirrhosis models indicates that MSCs can directly drive the polarization of BMDMs toward an M2 phenotype with enhanced phagocytic activity (150). This immunomodulatory effect is further mediated by MSC-derived exosomes, which deliver miR-148a to suppress the STAT3 pathway via targeting Kruppel-like factor 6 (KLF6) (151). Additionally, MSCs promote a phenotypic switch of monocytes from the profibrotic Ly6C^hi^ subset to the restorative Ly6C^lo^ subpopulation by secreting paracrine cytokines IL-4 and IL-10, thereby contributing to fibrosis resolution (152). The precise mechanisms by which MSCs sense the fibrotic niche to initiate this reprogramming remain an active area of investigation.

The functional interplay between mucosal-associated invariant T (MAIT) cells and macrophages is a dynamic, bidirectional process essential for tissue homeostasis and repair. While it is established that activated MAIT cells can orchestrate a proinflammatory macrophage phenotype, their role in resolution is increasingly appreciated (153). Evidence indicates that MAIT cell inactivation alters the transcriptional landscape of MoMFs, upregulating apoptotic genes (including members of the caspase family) in proinflammatory Ly6C^hi^ MoMFs while enhancing survival genes in proresolutive Ly6C^lo^ MoMFs. Furthermore, MAIT cells mediate metabolic reprogramming of the glycerophospholipid metabolic pathway in Ly6C^hi^ MoMFs, which facilitates the conversion of Ly6C^hi^ to Ly6C^lo^ phenotype. Both mechanisms promote scar resolution through sophisticated interactions with macrophages (154). A key unanswered question is precisely how MAIT cells initiate these distinct transcriptional and metabolic programs in different macrophage subsets.

In addition to the liver cells, the spleen can also orchestrate hepatic fibrosis through multiple mechanisms. Compelling data from CCl_4_-induced liver fibrosis reveal that splenic macrophages distally facilitate the secretion of CCL2 in hepatic macrophages via SOCS3 signaling, which subsequently induces the infiltration of circulating monocytes and the development of liver fibrosis in CCl_4_-induced liver fibrosis (155). On the other hand, splenectomy contributes to the infiltration and phenotypic switch of MoMFs toward a Ly6C^lo^ phenotype via the ERK1/2 signaling pathway in CCl_4_ and TAA-induced liver fibrosis (156). Spleen-derived lipocalin-2 (Lcn2), an antimicrobial protein, can modulate KCs activation and immune tolerance to mitigate fibrosis development in the above models (157). Further detailed research should be conducted to elucidate the intricate network of intercellular communications driving the pathogenesis and progression of liver fibrosis.

### 4.5 Metabolic reprogramming

**Figure 6:**
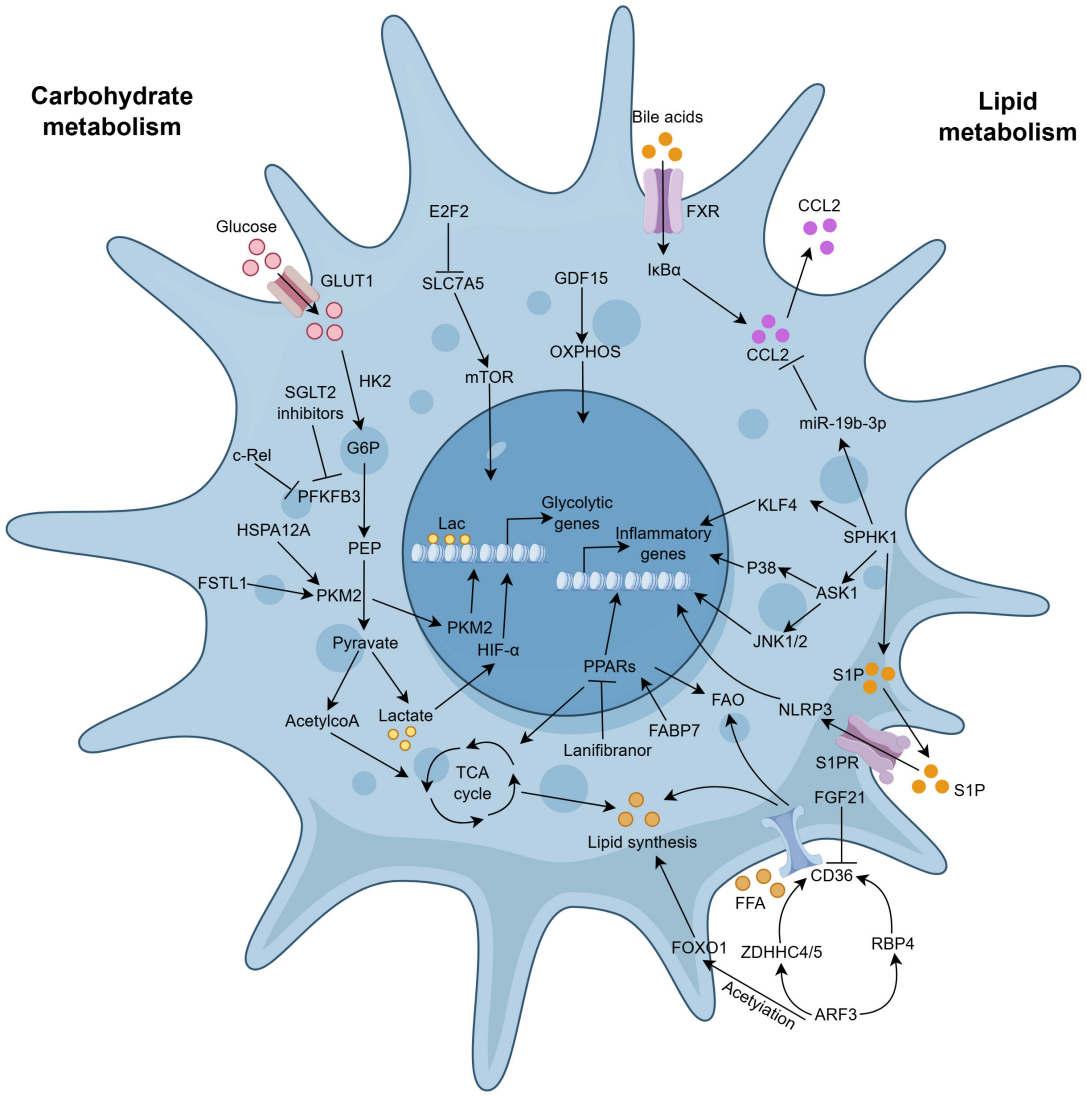
Metabolic reprogramming in hepatic macrophage activation during liver fibrogenesis. Metabolic reprogramming, such as carbohydrate metabolism, lipid metabolism, is responsible for macrophage activation. (1) Carbohydrate metabolism, M1 macrophage polarization mainly relies on aerobic glycolysis, while M2 macrophage polarization depends more on OXPHOS. GDF15 can preprogram the macrophages to commit to OXPHOS. PKM2 is a central enzyme for glycolysis. FSTL1 can bind and enhance the stability of PKM2. Both FSTL1 and HSPA12A can increase phosphorylation and nuclear translocation of PKM2. In contrast, ANXA5 inhibits phosphorylation and promotes PKM2 tetramer formation. PFKFB3 is another key activator of glycolysis. SGLT2 inhibitors and c-Rel can downregulate PFKFB3 in macrophages. (2) Lipid metabolism, CD36 is a key lipid transporter of hepatic macrophages. FGF21 can abolish HFCD-induced upregulation of CD36. ATF3 can increase RBP4 and Zdhhc4/5-mediated CD36 palmitoylation and block FOXO1 deacetylation to regulate gluconeogenesis. S1P is the most important lipid mediator, which can be catalytically synthesized by SPHK1. SPHK1 promotes macrophage polarization via the ASK1-JNK1/2-p38 signaling pathway and KLF4 SUMOylation. Activation of SPHK1 signaling contributes to macrophage recruitment by preventing miR-19b-3p-mediated inhibition of the CCL2–CCR2 axis. S1PR2/3 blockade downregulates the NLRP3 inflammasome and regulates BMDM activation. (3) Others, E2F2 downregulates the expression of SLC7A5 to mediate amino acid flux, resulting in enhanced glycolysis in a Leu‐mTORC1‐dependent manner. Bile acids activating FXR can repress KC activation and CCL2 release by upregulating IκBα. ATG5-mediated autophagy, Nup85-mediated CMA, and LAP are also associated with macrophage recruitment and polarization.

The local microenvironment usually changes after liver injury, which subsequently induces an altered metabolic state in macrophages, known as metabolic reprogramming, a process that critically governs macrophage polarization and the ultimate fate of liver fibrosis (**Figure 6**) (158).

#### 4.5.1 Carbohydrate metabolism

Recent advances in immunometabolism have established a strong link between macrophage activation and metabolic reprogramming. The classic dichotomy delineates a reliance on aerobic glycolysis for proinflammatory M1 activation, contrasting with oxidative phosphorylation (OXPHOS)-fueled anti-inflammatory M2 polarization. For example, the stress-responsive cytokine growth differentiation factor 15 (GDF15) has emerged as a potent modulator of macrophage immunometabolism. GDF15 deficiency reprograms macrophage metabolism toward OXPHOS, skewing polarization toward a profibrotic Ly6C^hi^ phenotype and exacerbating their infiltration in mice treated with CCl_4_ or a DDC diet (159).

The hypoxic microenvironment ensuing from acute and chronic liver injury is well established to drive metabolic reprogramming in macrophages, shifting their energy production from oxidative phosphorylation to aerobic glycolysis (160). Glycolysis provides intermediate metabolites and energy support for cell proliferation and phenotypic transformation during liver fibrosis. A pivotal player in this process is the M2 isoform of pyruvate kinase (PKM2), which functions as a central and targetable metabolic node orchestrating the transition to glycolysis (161). In murine models of MASLD, phosphorylation of PKM2 enhances glycolytic flux, promoting the polarization of macrophages toward a proinflammatory phenotype that exacerbates steatohepatitis and fibrosis, a process mediated in part by NLRP3 inflammasome activation (162). Importantly, the pharmacological PKM2 agonist ML265 has been shown to attenuate both macrophage activation and their profibrotic crosstalk with HSCs, underscoring the therapeutic potential of targeting this pathway (163). Follistatin-like 1 (FSTL1) is a key regulator that directly binds to PKM2, shielding it from ubiquitin-mediated degradation and thereby enhancing its cytoplasmic stability in hepatic macrophages from both human and murine fibrotic livers. In addition, FSTL1 promotes PKM2 phosphorylation and subsequent nuclear translocation, a metabolic switch that ultimately licenses proinflammatory macrophage polarization and enhances glycolytic flux. This FSTL1–PKM2 axis has been demonstrated to drive fibrosis progression across three murine models, including CCl_4_ injection, BDL, or a MCD diet (164). This paradigm of PKM2 chaperoning is further supported by the role of Homo sapiens heat shock 70 kDa protein family member 12A (HSPA12A), which also facilitates the nuclear translocation of PKM2, thereby reinforcing M1 polarization in a murine NASH model (165). In contrast, Annexin A5 (ANXA5) presents a counter-regulatory mechanism by interacting with PKM2 to inhibit its phosphorylation, thus promoting the formation of metabolically active tetramers and modulating macrophage activation in NASH (166).

6−Phosphofructo−2−kinase/fructose−2,6−bisphosphatase 3 (PFKFB3), another critical glycolytic activator, also functions as a regulatory nexus linking metabolic reprogramming to macrophage phenotype. This mechanistic insight is complemented by pharmacological evidence showing that sodium-glucose co-transporter 2 (SGLT2) inhibitors can attenuate NAFLD by downregulating PFKFB3, thereby suppressing glycolytic flux and altering macrophage polarization (167). Further solidifying PFKFB3’s pivotal position, research in CCl_4_-treated mice has revealed that the NF-κB subunit c-Rel orchestrates specific metabolic reprogramming in macrophages, driving them toward an M2 phenotype through the direct transcriptional control of PFKFB3 expression (168). Overall, growing evidence demonstrates the role of glycolysis in macrophage polarization; the upstream drivers of macrophage glycolytic reprogramming, however, are yet to be fully delineated. Potential sources include damaged hepatocytes (releasing DAMPs), activated hepatic stellate cells, and gut-derived metabolites; yet, definitive causal links and a hierarchy among these signals are lacking.

L-Lactate, the byproduct of glycolytic metabolism, has been found to mediate epigenetic changes in macrophages through a newfound lactylation modification, thereby regulating their phenotypic transformation (169). It has been proposed that M1 macrophages possess an intrinsic “lactate clock”, in which lactate can be enzymatically converted to lactoyl coenzyme A, facilitating histone H3K18 site (H3K18la) enrichment in the promoter region mediated by p300, which directly promotes the transcription of homeostatic genes and M1 polarization (170). Further evidence shows that overexpressed hexokinase 2 (HK2)-mediated glycolysis enhancement and elevated histone H3 lysine 18 lactylation (H3K18la) levels in liver macrophages drive macrophage polarization toward a proinflammatory M1 state, creating a self-amplifying loop sustaining inflammation in MASLD (171). Pharmacological intervention with salvianolic acid B functions to interrupt this circuit by downregulating lactate dehydrogenase A (LDHA) expression. This reduction in H3K18la levels attenuates M1 polarization and confers protection against CCl_4_-induced liver injury in mice (172). Beyond its role in inflammatory responses, histone lactylation has emerged as a novel regulatory layer that facilitates the activation of M2-type macrophages. Lactic acid promotes histone H3K27 acetylation, allowing the expression of an immunosuppressive gene program, including nuclear receptor subfamily 4 group A member 1 (Nr4a1) and arginase 1 (Arg-1). Consequently, macrophage proinflammatory function was transcriptionally repressed. Furthermore, the histone acetylation induced by lactic acid promotes a form of long-term immunosuppression (“trained immunosuppression”) (173). However, the complete landscape of lactylation and its specific targets in liver fibrosis remains incomplete. In particular, the identities and functional consequences of lactylation on nonhistone proteins are poorly understood.

#### 4.5.2 Lipid metabolism

Dysregulated lipid metabolism in macrophages is a pivotal driver of their polarization, thereby fueling the progression of MASH and liver fibrosis (174). Lipid synthesis is recognized as a mechanism that maintains the inflammatory phenotype of M1-like macrophages, whereas fatty acid oxidation (FAO) is required for M2-like macrophage polarization (175). Key regulatory nodes within this metabolic network present attractive therapeutic targets. For instance, inhibition of G protein-coupled receptor 84 (GPR84), a receptor for medium-chain fatty acids, attenuates the recruitment of neutrophils and MoMFs in murine models with MCD diet, CDAHFD diet, and CCl_4_ treatment (176). Similarly, targeting *de novo* lipogenesis through acetyl-CoA carboxylase (ACC) inhibition with WZ66 alleviates the activation of both KCs and HSCs in NASH models (177).

The lipid transporter CD36, highly expressed on liver macrophages, not only facilitates free fatty acid uptake but also actively participates in macrophage activation (178). Activating transcription factor 3 (ATF3) can increase Zdhhc4/5-mediated CD36 palmitoylation and block FOXO1 deacetylation to regulate gluconeogenesis in macrophages. In addition, ATF3 can improve hepatic macrophage glucolipid metabolism and reduce hepatocyte steatosis depending on retinol-binding protein 4 (RBP4) in the pathogenesis of MASH (179). Fibroblast growth factor 21 (FGF21) abolishes high-fat, high-calories diet (HFCD)-induced upregulation of CD36 in Kupffer cells, thereby preventing KC activation, collagen accumulation, and fibrogenesis (180). FGF21 also counteracts ferroptosis by promoting HO-1 ubiquitination and nuclear factor E2-related factor-2 (Nrf2) expression, offering protection against iron overload-induced injury and fibrosis (181). Pegozafermin, an analog of FGF21, has demonstrated efficacy in improving NASH fibrosis and severe hypertriglyceridemia. This research will advance into phase 3 clinical trials (182).

Sphingosine 1-phosphate (S1P), one of the most important lipid mediators derived from sphingomyelin metabolism, is catalytically synthesized by sphingosine kinase 1 (SPHK1). In BMDMs, SPHK1 promotes M1 and M2 macrophage polarization via apoptosis signal-regulating kinase 1 (ASK1)-c-Jun N-terminal kinase (JNK)1/2-p38 signaling pathway and Kruppel-like factor 4 (KLF4) SUMOylation, respectively (183). SPHK1 signaling activation in both HSCs and KCs exacerbates liver fibrosis by enhancing macrophage recruitment. This occurs through the disruption of the miR-19b-3p-mediated inhibition of the CCL2–CCR2 axis, as demonstrated in CCl_4_-induced murine models (184). Consistently, S1P receptor (S1PR) is responsible for proinflammatory macrophages activation and the development of liver fibrosis in methionine–choline-deficient and high-fat (MCDHF) diet-fed mice. S1PR2/3 blockade attenuates macrophage activation and accumulation by modulating the NLRP3 inflammasome in mice with BDL (185, 186).

In addition to lipid synthesis, FAO is intricately coupled to the functional state of macrophages, serving as a critical determinant of their activity (187). PPARs are essential regulators of fatty acid FAO in anti-inflammatory macrophages and are involved in the regulation of liver fibrosis (188). Lanifibranor, a pan-PPAR agonist, targets all PPAR isoforms and has been reported to drive hepatic macrophages to acquire an anti-inflammatory phenotype in experimental models of NASH induced by diet and chronic toxic injury via chronic CCl_4_ administration (189). Furthermore, following a phase IIb clinical trial, lanifibranor is now under investigation in phase III trials (190). Both PPAR-γ and PPAR-δ promote the differentiation of hepatic macrophages into an anti-inflammatory phenotype by regulating genes involved in fatty acid and cholesterol transport, as well as the TCA cycle. This transcriptional reprogramming enhances the lipid-handling capacity of macrophages, promotes cholesterol efflux, and supports OXPHOS, collectively driving a restorative macrophage polarization state (191). Consequently, deletion of either PPAR isoform in macrophages exacerbates hepatic steatosis and fibrosis. Accordingly, macrophage-specific deficiency in fatty acid-binding protein 7 (FABP7) reduces the levels of PPAR-γ, CCL17, and TGF-β, thereby impairing M2 polarization (192). Peroxisomes, the primary organelles for the β-oxidation of very long-chain fatty acids (VLCFAs), generate shorter-chain fatty acid derivatives that act as ligands for PPAR activation. Peroxisomal dysfunction in macrophages, particularly KCs, results in the accumulation of VLCFAs. These lipids subsequently function as DAMPs, activating the NLRP3 inflammasome and TLR signaling pathways, which drive macrophages toward a proinflammatory phenotype, promoting fibrosis progression (187).

Monoacylglycerol lipase (MAGL) plays a crucial role in catalyzing the hydrolysis of monoglycerides into glycerol and fatty acids. Genetic targeting of MAGL specifically in myeloid cells in murine models of toxic and biliary fibrosis results in reduced inflammation and fibrosis, as well as a shift in macrophage populations from a pro-inflammatory Ly6C^hi^ to an adaptive Ly6C^lo^ phenotype (193). Critically, the protective effects of myeloid MAGL knockout or pharmacological MAGL inhibition are dependent on increased autophagic activity in macrophages (61).

#### 4.5.3 Other metabolic ways

Amino acid metabolism reprogramming is increasingly recognized as a fundamental driver of macrophage polarization in liver fibrosis. E2F transcription factor 2 (E2F2), a transcription factor, is a significant regulator of proliferation, differentiation, and apoptosis. Myeloid‐specific E2F2 depletion upregulates the expression of the solute carrier family 7 member 5 (SLC7A5), enhancing the influx of leucine and other amino acids, which activates the mTORC1 pathway, driving a metabolic shift toward glycolysis and promoting a proinflammatory macrophage phenotype that exacerbates MASH progression (194).

Bile acids are emerging as critical modulators of macrophage function. The nuclear receptor Farnesoid-X receptor (FXR) in KCs can polarize macrophages toward an anti-inflammatory phenotype. The synthetic bile acid and FXR agonist obeticholic acid (OCA) can repress KC activation, potentially by inhibiting the NF-κB pathway, which is achieved through the upregulation of its cytoplasmic inhibitor, inhibitor kappa B alpha (IκBα), and the downregulation of the proinflammatory chemokines CCL2 in response to TAA administration (195).

Autophagy, including macroautophagy, microautophagy, and chaperone-mediated autophagy (CMA), is a lysosomal degradation pathway of cellular components that displays anti-inflammatory properties in macrophages. Its role in hepatic fibrosis has been implicated by studies showing that autophagy inhibits M1 macrophage polarization and EV secretion, thereby attenuating disease progression in CCl_4_-induced models (196). Further study corroborates this concept: autophagy deficiency promotes M1 macrophage polarization via ATG5 repression in TAA-induced murine models (197). Similarly, impaired CMA in macrophages aggravates liver-targeted recruitment of monocytes and exacerbates fibrosis, which can be attenuated through inhibition of the substrate nucleoporin 85 (Nup85) in NASH mice (198). LC3-associated phagocytosis (LAP) is a noncanonical form of autophagy; pharmacological inhibition of LAP components or genetic disruption of LAP shifts the monocyte/macrophage phenotype to a proinflammatory phenotype. This shift aggravates CCl_4_-induced liver injury and fibrosis by dampening the protective FcγRIIA/SHP-1 inhibitory immunoreceptor tyrosine-based activation motif (ITAMi) signaling pathway (199).

Though it is increasingly recognized that metabolic reprogramming is crucial for macrophage polarization, a critical yet underexplored layer of complexity is whether macrophages from different origins (e.g., Kupffer cells versus monocyte-derived) and belonging to distinct functional subsets (e.g., LAMs) undergo unique, subset-specific metabolic rewiring. Furthermore, can insights from metabolic reprogramming be exploited to develop novel dietary or pharmacological strategies?

## 5 Conclusions

Hepatic macrophages exert a crucial yet paradoxical role in the progression and regression of liver fibrosis. Consequently, targeting these cells has emerged as a promising therapeutic avenue. Strategies aimed at orchestrating macrophage polarization, impairing monocyte recruitment, and modulating the immune microenvironment—through metabolic reprogramming, key signaling pathways, and noncoding RNAs—are under intense investigation, with several candidates advancing into clinical trials. A deeper dissection of the mechanisms governing macrophage differentiation and recruitment is therefore imperative, as it holds the key to unlocking novel and effective antifibrotic therapies for the future.

However, significant challenges and knowledge gaps remain. The field must move beyond the simplistic M1/M2 paradigm and fully embrace the heterogeneity of macrophage states defined by single-cell technologies. Key unanswered questions include: How can we therapeutically target specific profibrotic macrophage subsets (like SAMs or LAMs) without compromising their essential homeostatic and restorative functions? Can insights from metabolic reprogramming be harnessed to develop effective dietary or pharmacological interventions? Whether these mechanisms engage in crosstalk, forming an interconnected network?

Future research should prioritize the integration of multiomics data from well-characterized human cohorts and refined animal models to bridge species-specific differences. Translating these mechanistic discoveries into novel therapeutics, potentially through combination therapies and cell-specific targeting strategies, holds the key to effectively halting or reversing liver fibrosis in patients.
